# Supplementary material for: Saccharomyces boulardii (CNCM I-745) improves intestinal damage in sepsis by remodeling intestinal flora structure
Source: Front Cell Infect Microbiol. 2025 Jul 31;15:1602792. doi: 10.3389/fcimb.2025.1602792 (PMC12350329; doi:10.3389/fcimb.2025.1602792)
Supplement: Supplementary file 1 [file DataSheet1.zip › summary/summary/4_Alpha_diversity/1_rarefaction_curves/Group/Alpha_Diversity.RareCure.Chao1.Group.pdf]

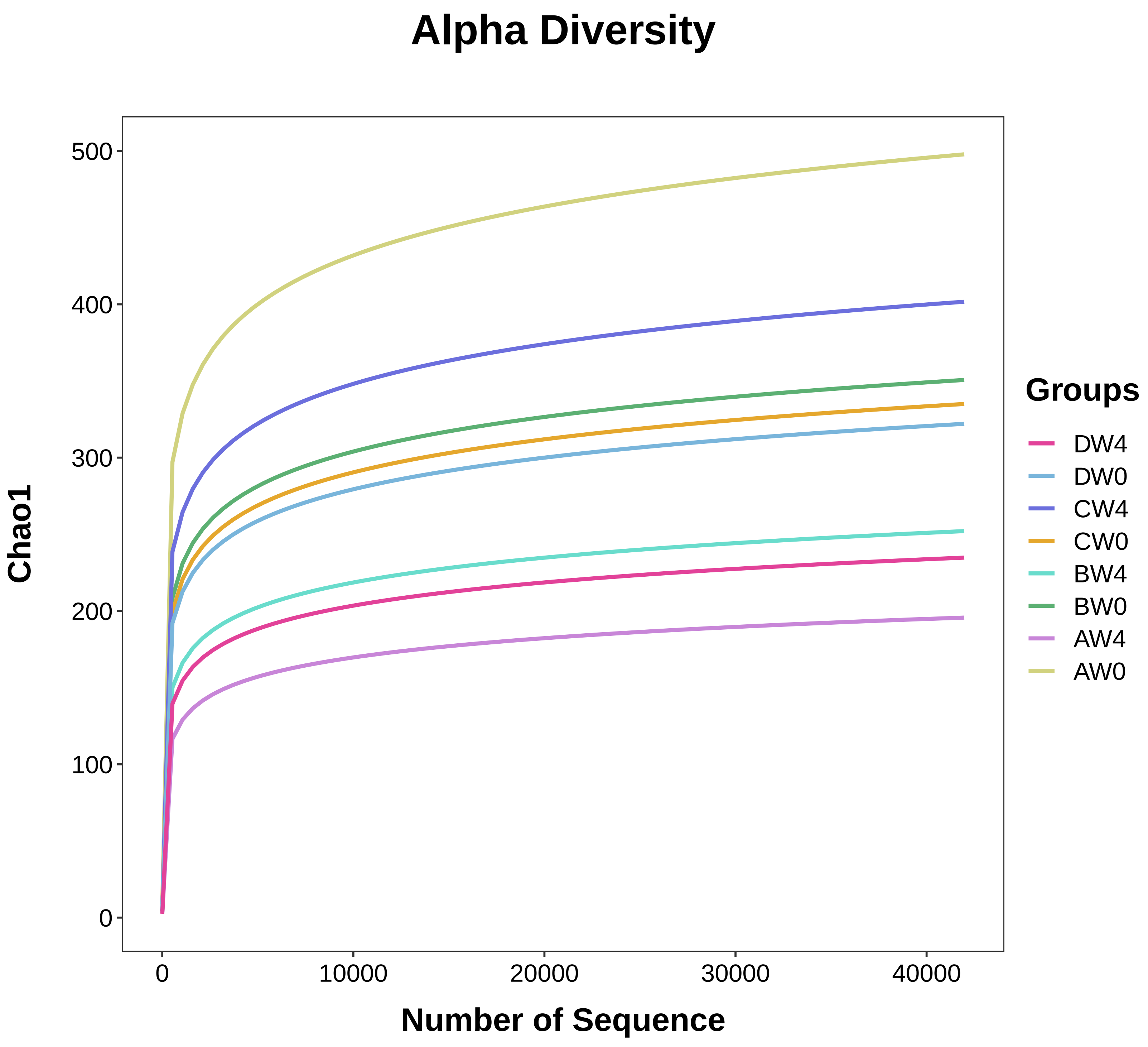
Alpha Diversity
Chao1
0
100
200
300
400
500
0
10000
20000
30000
40000
Number of Sequence
Groups
DW4
DW0
CW4
CW0
BW4
BW0
AW4
AW0
